# Research on Secure Debugging Interaction of Sensor Nodes Based on Visible Light Communication

**DOI:** 10.3390/s21030953

**Published:** 2021-02-01

**Authors:** Yuanchu Yin, Jiefan Qiu, Zhiqiang Li, Mingsheng Cao

**Affiliations:** 1 College of Computer Science and Technology, Zhejiang University of Technology, Hangzhou 310023, China; 2111912167@zjut.edu.cn (Y.Y.); qiujiefan@zjut.edu.cn (J.Q.); 2 College of Life Science and Technology, Central South University of Forestry and Technology, Changsha 410018, China; lizhiqiang@csuft.edu.cn; 3 School of Computer Science and Engineering, University of Electronic Science and Technology of China, Chengdu 610054, China

**Keywords:** sensor nodes, debugging, visible light communication, instruction, modulation

## Abstract

When a wireless sensor node’s wireless communication fails after being deployed in an inaccessible area, the lost node cannot be repaired through a debugging interaction that relies on that communication. Visible light communication (VLC) as a supplement of radio wave communication can improve the transmission security at the physical layer due to its unidirectional propagation characteristic. Therefore, we implemented a VLC-based hybrid communication debugging system (HCDS) based on VLC using smartphone and sensor node. For the system’s downlink, the smartphone is taken as the VLC gateway and sends the debugging codes to the sensor node by the flashlight. To improve the transmission efficiency of the downlink, we also propose a new coding method for source coding and channel coding, respectively. For the source coding, we analyze the binary instructions and compress the operands using bitmask techniques. The average compression rate of the binary structure reaches 84.11%. For the channel coding, we optimize dual-header pulse interval (DH-PIM) and propose overlapped DH-PIM (ODH-PIM) by introducing a flashlight half-on state. The flashlight half-on state can improve the representation capability of individual symbols. For the uplink of HCDS, we use the onboard LED of the sensor node to transmit feedback debugging information to the smartphone. At the same time, we design a novel encoding format of DH-PIM to optimize uplink transmission. Experimental results show that the optimized uplink transmission time and BER are reduced by 10.71% and 22%, compared with the original DH-PIM.

## 1. Introduction

The wireless sensor network is composed of decentralized and self-organized sensor nodes. Once the network deployment, it is difficult to maintain the nodes, especially the nodes are deployed in an inaccessible area. The reparation and debugging of sensor nodes have to rely on the built wireless network infrastructure. Actually, data transmission during debugging interaction between sensor nodes and the cloud is mainly completed by radio wave communication (RWC) whose frequency is from 10 kHz to 3000 GHz, such as 2.4 GHz (Zigbee and Wi-Fi) or 800 MHz (GPRS), etc. Due to the broadcast nature of RWC, the transmitted data are vulnerable to eavesdropping. Additionally, by RWC way is difficult to debug the lost sensor nodes which cannot contact with other sensor nodes or cloud. In these lost sensor node cases, some failures in wireless communication of sensor nodes are related to malicious intrusion. That means it is no longer safe to transmitting any sensitive data (such as debugging information or codes) by RWC way. Even once the intruders obtain the debugging codes, they can further inject the malicious codes into the sensor node and continuously intercept more sensitive data.

The previous studies rarely consider in improving the security of debugging interaction from the physical level of communication. In practice, most developers have to abandon these lost or abnormal nodes and replace them with backup nodes [1,2,3]. Meanwhile, visible light communication (VLC) adopts unidirectional propagation different from RWC with broadcast way. This unidirectional propagation guarantees intruders difficult to eavesdrop on and helps to reduce security risk. Therefore, VLC can provide more secure data transmission at the physical level. In addition, VLC can be taken as supplemental communication means of RWC. Once the sensor node is not able to recover wireless communication, some debugging information can be back to the cloud by VLC. This debugging information helps developers to analyze the lost reason and possibility of malicious intrusion and further improve the overall security of the current network.

Current VLC studies generally require dedicated visible light sensing components or modules. For example, Fan et al. [4] applied VLC to the access control system by introducing a special VLC circuit. The circuit composes of the photodiode, amplifier, comparator, and MCU. In order to increase transmission rate and solve the problem of multi-channel transmission, Wang et al. [5] designed a duplexing indoor communication system that needs dedicated RGB light emitting diodes (LED), low pass filters, electronic amplifiers. Because VLC components are sensitive to environmental optical noise, Adiono et al. [6] proposed a solution to reduce the influence of optical noise. This solution requires an analog filter as the front-end receiver to preprocess the received light signal. Such extra hardware modifications for VLC are hard to be afforded by low-cost sensor nodes. That also becomes a bottleneck of the VLC applying in the sensor node device. Therefore, we need to explore how to apply VLC in low-cost or Commercial Of-The-Shelf (COTS) devices.

In recent years, with the development of visible light technology, the sensor node (such as TelosB, Micaz) and smartphone equip various visible light components, such as LED, camera, and ambient light sensors. These components provide a potential capacity for VLC. In this paper, we implemented a VLC-based hybrid communication debugging system (HCDS) applied in smartphones and sensor nodes. In the system, the smartphone is taken as a VLC-based gateway that connects the sensor node with the cloud and completes debugging interaction. In the system uplink, debugging information from the sensor node is transmitted by the node’s LED and received by smartphone’s optical camera. In the system downlink, debugging codes are transmitted by the smartphone’s flashlight and received by the node’s ambient light sensor. Obviously, although both uplink and downlink are realized by VLC, the different signal receivers make the difference in processing light signals.

In the system downlink, the ambient light sensor of the sensor node has a sensing latency. This latency limits the minimum width of the light pulse and causes a low sensing resolution which decreases data transmission efficiency. For this, we optimize system downlink transmission from the source and channel coding, respectively. With respect to source coding, we analyze the debugging codes and find a part of the operand (such as a base address) that can be compactly represented by a bitmask. With respect to channel coding, we propose an overlap dual-header pulse interval modulation (ODH-PIM) optimized for smartphone’s flashlight. Like DH-PIM, ODH-PIM also adopts the width of the low power to represent a symbol and introduce a flashlight half-on state as an overlapping mark. The overlapping mark enhances the representation ability of a symbol and further improves transmission efficiency.

In system uplink, we adopt the optical camera communication (OCC) for the debugging information transmission. The previous studies have applied DH-PIM in OCC. Due to the poor performance of COTS cameras of smartphones, directly applying DH-PIM in the camera increases the bit error rate (BER). Therefore, we modify the symbolic representation of DH-PIM and optimize the demodulation procedure to improve transmission stability.

Finally, we conducted a series of experiments in HCDS and verified our methods above from compression rate, bit error rate, and transmission time. The experiment results illustrate that our compression method reduces 80% of redundant codes. Simultaneously, the ODH-PIM owns less transmission time than the original DH-PIM at the cost of an extra 17.36% energy overhead. Moreover, the modified DH-PIM can effectively reduce the average 29% BER for the uplink transmission.

The rest of this paper is structured as follows: Section 2 shows related work; Section 3 gives the overview of the VLC-based HCDS; Section 4 presents the optimization in transmission efficiency of the system downlink; Section 5 presents the optimization in transmission stability of the system uplink; Section 6 describes experiments and evaluates system performance, and Section 7 closes our work with a conclusion.

## 2. Related Work

Previous security efforts for sensor network debugging pay more attention to general attack and excessive security overhead. For example, Sluice’s [7] verification mechanism provides authenticity and integrity through a hash-chain construction that amortizes the cost of a single digital signature over an entire update. Hyun et al. [8] presented the Seluge which provided security protections for code dissemination by efficiently using cryptographic primitives. Park et al. [9] presented a recovery method for lost packet, which used the redundant hash scheme and page digest scheme. In the Sreluge [10], the author employs a neighbor classification system and a time series forecasting technique to isolate polluters. In recent years, machine learning (ML) and deep learning (DL) techniques have also been applied in enhancing the sensor network security. Doshi et al. [11] illustrated that using network behaviors to help feature selection can result in high accuracy detection of DDoS with a variety of ML algorithms. Diro et al. [12] designed a distributed attack detection system based on DL to discriminate attacks from benign traffic. Some ML-based approaches are also used to detect and eliminate malicious nodes. Rathore et al. [13] used biological inspirations and ML to identify fraudulent nodes. An enhanced trust model based on the radial base artificial neural network (RBANN) was presented by Yasin et al. [14] to predict each node’s future behavior and detect malicious nodes. Yoon et al. [15] provided a DL-based approach to verify the trustworthiness of sensors by considering the sensor data only. These studies focus on applying ML and DL to identify the attack or faulty sensor node and give little consideration to improving the security of communication, not to mention enhancing the security from the physical level.

With respect to VLC, most studies focus on optimize the modulation method to improve the performance of VLC. Ghassemlooy et al. [16] designed the digital pulse interval modulation (DPIM), which uses non-symbol synchronization to simplify the receiver structure. However, DPIM’s non-uniform symbol structure may cause problems of buffer overflow in a network environment. Subsequently, DH-PIM [17] is proposed to improve transmission stability by adding a built-in frame synchronization. Ali et al. [18] combined pulse position modulation (PPM) and pulse shape modulation (PSM) to the proposed PPSM, which increases the number of pulses and bandwidth to keep a low BER at a high transmission rate. Deng [19] proposed MPPM, which can keep a fixed average light intensity to achieve different levels of dimming control, but it cannot support fine-grained dimming levels. MH-PIM [20] employs different symbol headers to represent an input sequence with less frame length. In other the hand, with the development of CMOS cameras, researchers proposed optical camera communication (OCC) based on rolling shutter technology which extends VLC to the optical camera. Roberts et al. [21] optimized three OCC modulations: On-Off Keying (OOK), variable PPM, and color-shift keying (CSK) to solve the problem of dimming control. They [22] also introduce an under-sample in OCC for demodulate high-frequency OOK signals. CSK-CDMA [23] is proposed by Chen et al., who apply CSK and code-division multiple-access (CDMA) simultaneously to allow multiple users to access the network. Luo [24] proposed UPSOOK that represents different data by phase shift and realized non-flickering communication. Marshoud et al. [25] proposed a novel power-domain multiplexing based on optical asymmetric modulation (OAM) scheme which is used to transmit high-order modulation signals over VLC channels. Nguyen et al. [26] gave the sequence number (SN) to reduce the impact of frame rate variation.

Most of the above studies are based on dedicated optical camera or light sensor. Due to the limits of the resource and energy of sensor nodes, it is difficult to directly implement complex coding and modulation methods. On the other hand, there is not available VLC method directly applying in debugging interaction of sensor nodes. Therefore, we present VLC-based hybrid-communication debugging system (HCDS) for low-cost sensor nodes and COTS smartphone and propose appreciate coding and modulation methods for the HCDS.

## 3. Overview of Hybrid Communication Debugging System

**Figure 1:**
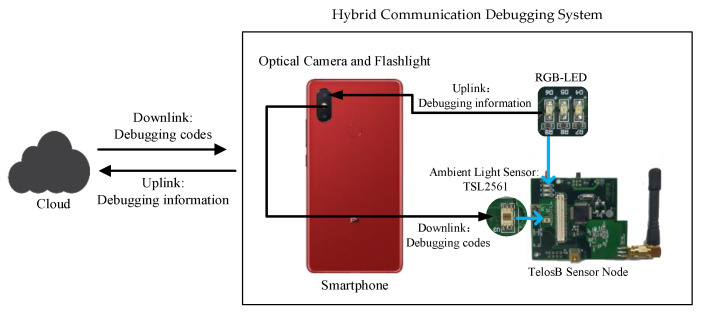
System overview.

To repair the wireless communication lost node, we designed HCDS based on VLC. As shown in Figure 1, the system consists of a COTS smartphone and a low-cost sensor node. The uplink and downlink of HCDS employ different visible light signal receivers. In system downlink, a regular ambient light sensor equipped by sensor node is used to sense continuous light pulses which debugging codes are modulated into. In system uplink, the optical camera as a standard configuration of the smartphone is taken as the light signal receiver. The camera adopts rolling shutter technology to capture several light pulses in one picture and further realize the optical camera communication. Finally, the flashlight and LED can be taken as an available light signal transmitter.

Debugging codes are commonly generated by the cloud, which executes cross-compilation for different types of sensor nodes. The smartphone as a VLC gateway collects the feedback debugging information from the sensor node and sends the debugging codes to it. The large memory space of the smartphone allows frequently-used debugging codes to be stored in the smartphone. Therefore, after the smartphone receives the sensor node’s debugging information, it confirms whether corresponding debugging codes are available locally. If so, the smartphone sends the local debugging codes directly through VLC; otherwise, it forwards the debugging information to the cloud and requests new debugging codes by regular radio wave communication.

Actually, HCDS is applied in an extreme situation in which the node has been malicious intruded or lost, and by normal wireless communication cannot complete a debugging procedure in safe way. At the same time, the system does not require any modification in the hardware of COTS smartphone and sensor node. Based on the above, the one-to-one synchronous communication mode and low transmission rate of HCDS can be acceptable to only transmit essential relevant-debugging data, unlike existing researches which can employ dedicated devices to achieve high transmission rate and realize complex coding scheme for broadcast or multicast [27,28,29,30]. In the other hand, limited to the performance of the VLC components equipped by COTS devices, the communication range of HCDS is difficult to effectively improve by software-level optimization. In practice, maintainers can carry the portable smartphone close to the target sensor node and control the communication range for completing the debugging integration. With respect to sensor nodes deployed in inaccessible areas, the drone can be taken as VLC gateway to execute debugging task. We will study this debugging scenario in our future work.

## 4. System Downlink: Transmission Efficiency Improvement

The system downlink transmits a large number of binary codes to repair or debug the sensor node. However, the sensing latency and low resolution of ambient light sensors result in low transmission efficiency of the overall codes. To this end, we employ two compression methods within the source and channel coding.

### 4.1. Source Coding: The Binary Instruction Compression

Since the number of transmitted binary codes is large, the transmitting procedure is overlong for debugging interaction due to the low resolution of the low-cost light sensor. Thus, it is necessary to minimize the size of codes by compressing them. The traditional Huffman compression has a high compression ratio, but an extra dictionary must be locally stored in the sensor node and occupies a precious memory resource. The ZIP compression is also not suitable for low-cost sensor nodes due to the large computation overhead. Therefore, we attempt to analyze the characters of binary codes and propose a targeted compression method that removes the redundant data existing in each binary instruction. In our method, without any dictionary, our method complete codes compression in sensor node at low computation overhead.

Actually, in most debugging sensor node cases, transmitting the entire program image is unnecessary. In our debugging interaction, we repair the local program by updating each function [31]. One function consists of several instructions which finally compress into compressed binary codes. The specific instruction is relative to MCU type. Take MSP430 as an example. It is equipped in TelosB node and uses a 16-bit instruction set that contains 12 double-operand instructions, seven single-operand instructions, and eight jump instructions.

**Figure 2:**
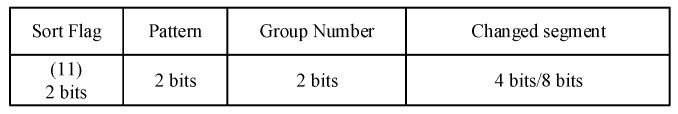
Operand sequence compression coding format.

One instruction can be divided into opcode and operand. The opcode is used to differentiate instruction and difficult to be compressed. In contrast, the operands existing many continuous repetitive bit sequences. Thus, we apply a bitmask to compress operand bit sequences. The operand bit sequences of each instruction is usually a register address or a memory address. And an all-zero sequence and these operand sequences might differ in just a small number of bits. We can use a bitmask to find these different data. There are two additional fields, called mask location and changed segment, which indicate the position of the mask and the different data compared to the all-zero sequence. They can be calculated by doing an OR operation between the all-zero sequence and every operand sequence. Moreover, the mask location is further grouped to facilitate the representation. The pattern and the group number represent the mask locations where changed segments will replace the data. The operand sequence encoding format as it shows in Figure 2. The sort flag must be “11” to indicate that this is an operand sequence. Next, we will introduce the schemes adopted by each pattern shown in Table 1.

**Figure 3:**
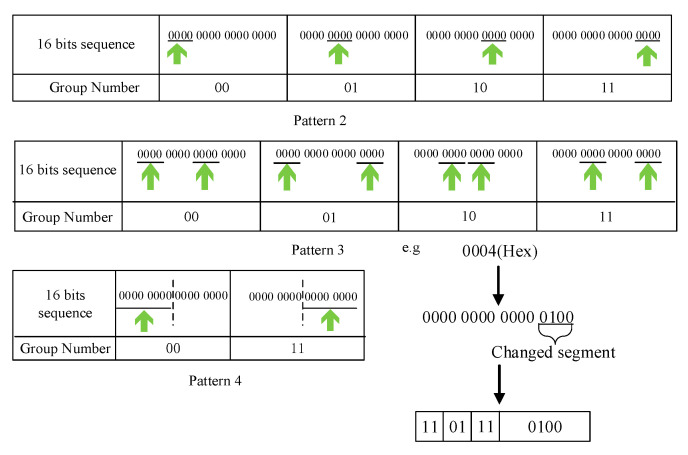
Pattern 2–4 diagram and compression example.

In pattern 1, it means the original data is not compressible and will be transmitted directly. Both pattern 2 and pattern 3 divide 16 bits of data into four groups. Each group has 4 bits in pattern 2 and pattern 3. Pattern 4 divides 16 bits of data into two groups; each group has eight bits. Additionally, when the group number in pattern 4 is “10”, it indicates all-zero data. The diagram of pattern 2 to pattern 4 is shown in Figure 3. Actually, our compression method for binary instruction mainly involves search operation for repetitive parts and replacement operation for bit-level compression and, thus, the complexity of the compression algorithm is O(N). We also test the compression time to prove the efficiency of this compression algorithm in Section 6.1.

### 4.2. Channel Coding: Overlap DH-PIM

**Figure 4:**
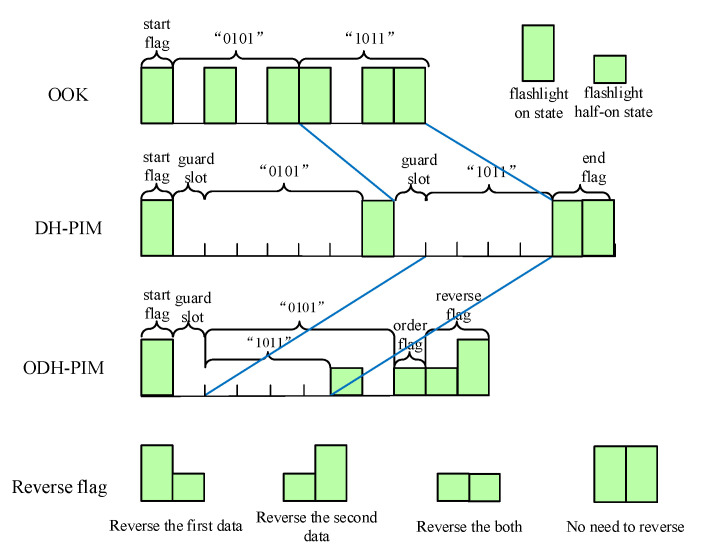
Three modulation comparison. Bit series “01011011”.

With respect to the channel coding, the minimum width of light slot pulse is limited by sensing latency, and the transmission time is extended. For this, we attempt to reduce the number of slots representing one DH-PIM symbol. By experiment, we found that ambient light sensor cannot measure a short-duration light pulse and output a lower value than actual value due to the sensing latency. We leverage this find to mark a new flashlight half-on state from the On/Off state. Further, we propose the overlap DH-PIM (ODH-PIM) by introducing a flashlight half-on state. DH-PIM is an isochronous pulse time modulation in which data is encoded as discrete slots between adjacent pulses. A symbol that encodes *M* bits of data is represented by k low power slots and followed by one constant power slot, where 0 ≤ k ≤ (L/2) − 1 and L = 2M. For example, the number “0100” has four low power slots and one constant power slot. The binary data “1011” is a radix-minus-one complement of 0100 which has four slots of low power and two slots pulse of constant power. If M = 4, a symbol of the DH-PIM contains four bits of data, but the ODH-PIM can compress 8 bits of data into one symbol because two data can overlap. As shown in Figure 4, the data “1011” was overlapped with the data “0101”, and they share the front four low power slots. Because different data have different slots, when two data are overlapped together for modulation, the flashlight half-on state plays the role to differentiate them. As shown in Figure 4, after representing the contents of two data, a slot is introduced as an order flag. If the order flag is a constant power slot, the data with less slot precedes the other one. If the order flag is a slot of flashlight half-on state, it indicates that the order of the two data needs to be changed. Then, there are four cases of the reverse flag to determine which data needs to calculate complement.

At the beginning of downlink transmission, a start signal is sent to the sensor node from the smartphone by flashlight, and it is also used to synchronize two devices. During code transmission period, the node as a receiver guarantees the integrity of the frame by countering non-low power slots (flashlight on and half-on state), because the integrated frame has the same slots (five slots for ODH-PIM). Once the node detects a wrong number of non-low power slots in a received frame, it will turn on local LED to tell the smartphone that a frame error happened and requires it to retransmit the frame. During downlink transmission, the smartphone also activates the camera to receive the frame error message and then retransmit the corresponding frame.

## 5. System Uplink: Transmission Stability Improvement

We apply optical camera communication (OCC) in system uplink transmission. In our system, the smartphone’s camera is taken as a receiver. The slow scanning speed of rolling shutter camera makes the BER boosting, because it is difficult for the camera to distinguish the bright and dark stripes when LED flicker speed is high. In addition, the camera is not sensitive to the constant power slot under some outdoor environments in which strong background light exists.

**Figure 5:**
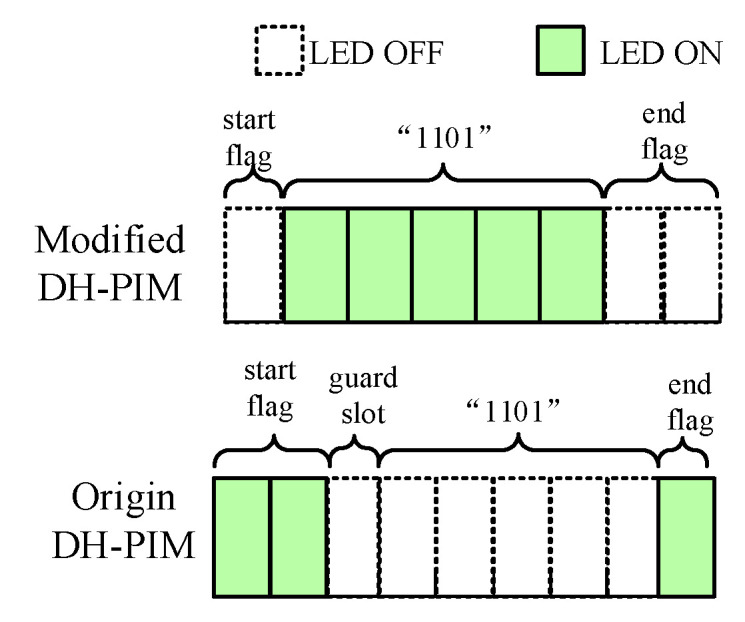
Comparison before and after modification of DH-PIM.

DH-PIM is also commonly applied in OCC. From discussion in Section 4.2, it relies on the number of low power slots to represent one symbol and one constant power slot to segment two symbols. That effectively reduces LED status toggles and the demand of scanning speed. We modify encoding format of DH-PIM, because a single constant power slot formed by bright stripe is not easy to detect in image. The format is shown in Figure 5. In this format, a low power slot is used as the frame head, which segments symbols, followed by constant power slots to represent a symbol. One symbol contains four bits of data. Two low power slots in the frame end indicate complemented operation in data, and one slot means not.

The demodulation needs to calculate the number of constant power slots by measuring bright stripes’ width in the image captured by camera. The first dark stripe as the start flag contains only one low power slot, so its width can be used to calculate the number of constant power slots.

**Figure 6:**
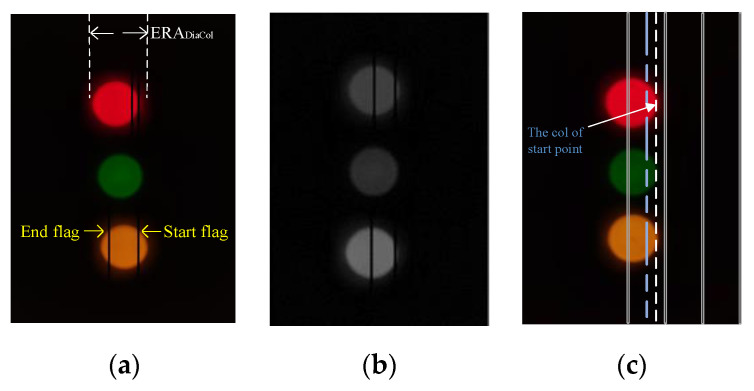
(**a**) Photos captured by the camera. (**b**) Image with only Y channel. (**c**) Successive approximation method.

After measuring the width of start flag and the following bright stripes, the receiver calculates the number of constant power slots. Additionally, sum widths of all stripes cannot exceed the effective receiving area (ERA) in an image which is relative to exposure time. Otherwise, a symbol cannot be completely obtained in the image. If we define the time of one slot is *T_slot_* and adopt the column scanning, the range of *T_slot_* is:

(1) $$\left[ScanCol_{Time},\frac{ERA_{DiaCol}\cdot ScanCol_{Time}}{SYB_{NumSlots}}\right]$$

where *ERA_DiaCol_* respectively represent the number of pixel columns owned by one image and occupied by ERA as shown in Figure 6a. *ScanCol_time_* is the time to finish scanning single column. A *ScanCol_time_* is the maximum number of slots required for one DH-PIM symbol.

For sensor node, three LEDs are available for OCC. The green LED is used as the synchronization signal. When the dark stripe appears in this LED, it means that ERA cannot display a complete symbol, and it is necessary to resynchronization. The rest of two LEDs are used to transmit data.

For the camera, we optimize the image processing from two sides to improve demodulation efficiency. Firstly, applying YUV-color space reduces the amount of data and improves image processing speed. The Y channel contains the gray information of image, as shown in Figure 6b. Based on Y channel, there is enough information to distinguish the bright and dark stripes for demodulation of DH-PIM. Secondly, quickly detecting a start pixel point of ERA can increase the transmission rate. During image processing, we apply the successive approximation method in column scanning to obtain the start point shown in Figure 6c. In the initialization of successive approximation, each column is scanned at an interval of *K −* 1 columns; in each scanned column, each pixel point is sampled at an interval of K pixel points. K is smaller than *ERA_DiaCol_*. Once we sample a bright pixel point in the *nK*th column, we record the number k of all bright pixel points in this column and then rollback column scanning process to the (*n/2*)*K*th column. In the (*n/2*)*K*th, each pixel point is sampled at interval of *k/*2 pixel points. If a bright point is sampled, record the number *k* of all bright pixel points in this column and then rollback column scanning process to the (*n/*4)*K*th column; else scan the (*n* × 3/4)*K*th column. Repeat above process and obtain the start point with (*log*2*K + Imagecol/K*) times of scanning column at most. *Image_col_* denotes the number of pixel columns in one image.

## 6. Evaluation

Based on the Android operation system and general sensor nodes, we realize the VLC-based HCDS using a COTS Android smartphone and a popular sensor node TelosB. In this system, smartphone need to be equipped with a flashlight, optical camera, and its installed Android support for Camera2 API. The TelosB owns RGB-LED and ambient light sensor TSL2561.

### 6.1. Transmission Rate under Different Debugging Cases

In downlink, we conduct a series of experiments about updating code blocks from five different functions shown in Table 2. These blocks are stored as binary files. The timing and LS Ctrl function relative to controlling sensor node. Bubble sort, Dijkstra, and Horspool are all sort algorithms.

**Figure 7:**
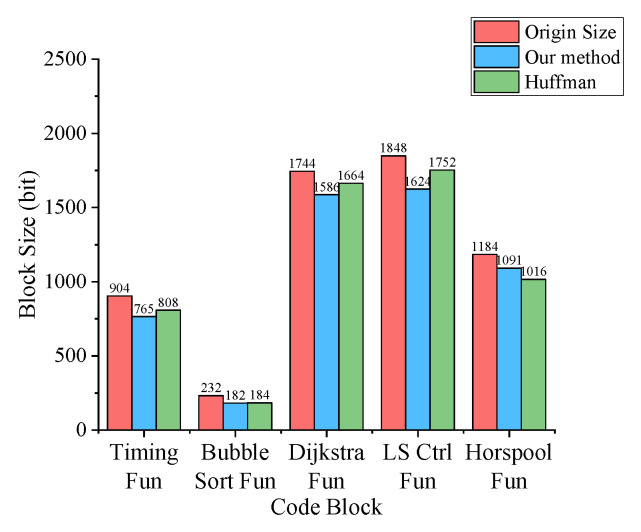
Compressed code size.

As shown in Figure 7, we compressed each of the five code blocks with traditional Huffman and our method, and Table 2 gives the compression rate and time of the five blocks with our compression method. After using our compression method, the code block size is reduced at least 8%, especially in Timing Fun with 16% reduction. From Figure 7, the average compression rate of our method has reached 86.8%. The reason is that the difference of operands of instructions makes binary codes appear irregular, and using Huffman is difficult to compress these irregular codes. In addition, Huffman needs to maintain a dictionary in the sensor node and occupy the precious RAM of the node. And if the sensor node does not have a corresponding dictionary, an additional dictionary will be transmitted by downlink, which reduces the transmission efficiency.

**Figure 8:**
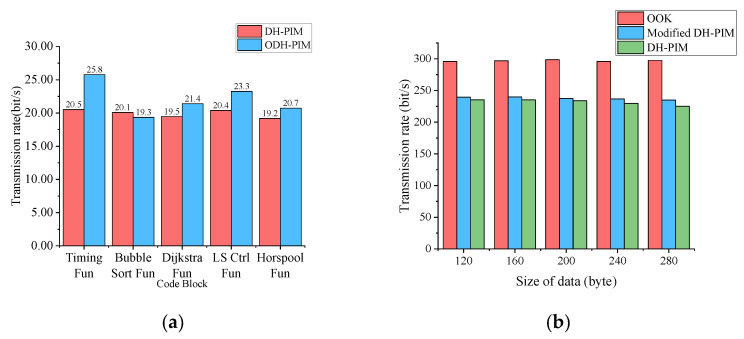
(**a**) Transmission rate in downlink. (**b**) Transmission rate in uplink.

The transmission rate of ODH-PIM is given in Figure 8a. We compared ODH-PIM with DH-PIM. In order to neatly compares, these two methods are used to transmit origin code without compression. In most cases of transmitting code blocks, our method is better than DH-PIM. Especially in the Timing Fun case, ODH-PIM is 25.8% faster than DH-PIM because ODH-PIM can effectively reduce a symbol’s length. In the Bubble Sort Fun case, we found that ODH-PIM needs more slots when a large number of consecutive zero existing in binary codes, and the transmission rate of ODH-PIM remains about 3.9% below DH-PIM.

In the uplink, we test OOK, DH-PIM, and modified DH-PIM by transmitting feedback data in five different debugging cases. It is obviously that the amount of transmitted data using DH-PIM cannot exceed using OOK under same slots. As shown in Figure 8b, the transmission rate of OOK is faster than DH-PIM. The modified DH-PIM has a slightly higher transmission rate than DH-PIM, and its BER is significantly lower than that of DH-PIM.

**Figure 9:**
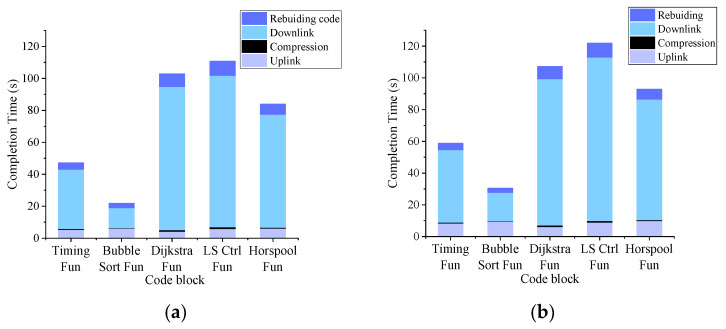
(**a**) Completion time in indoor environment (650 lux); (**b**) completion time in indoor environment (2100 lux).

Figure 9 shows the debugging completion time, which consist of downlink/uplink transmission, compression and local rebuilding time. We complete VLC-based debugging interaction under outdoor and indoor environments with different ambient light intensity. The whole procedure of debugging interactions includes: (1) The node sends the message “Update: xx fun” to the smartphone; (2) the smartphone firstly compresses the code block of the corresponding function (the size of code blocks is shown in Table 2), and then transmits the compressed data to the sensor node; (3) the sensor node decompresses the received data and locally rebuilds the program image. As shown in Figure 9, the most time-consuming process is the transmission of the debugging codes. In contrast, the code block’s compression occupies a small time period due to the computing power of smartphone. The debugging completion time in the indoor environment is 15% less than the time in the outdoor environment on average. Ambient light impacts uplink/downlink transmission and increases frame errors. To deal with frame errors requires extra retransmitting frame and prolong uplink/downlink transmission in outdoor environment. Moreover, the rebuilding time is relative to the size of the debugging code. The size of debugging codes is smaller, the rebuild time is shorter.

### 6.2. Testing BER under Different Angle and Distance

The bit error rate is an important fact to evaluate performance of transmission system. We conduct a series of experiments about the influence of angle and distance on BER.

**Figure 10:**
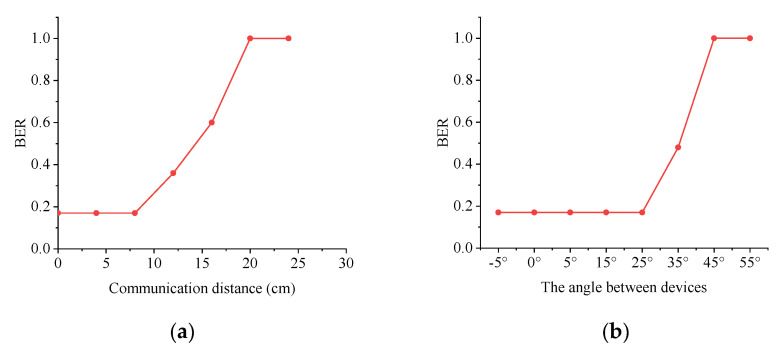
(**a**) BER under different distances in downlink. (**b**) BER under different angles in downlink.

Different distances between the flashlight to the ambient light sensor result in different light intensities captured by the sensor in the downlink. Figure 10a,b demonstrates the BER of ODH-PIM with different angles and distances. From Figure 10a, it is obvious that BER is very high when the transmission distance exceeds 20 cm. The reason is that the sensor is difficult to detect the flashlight half-on state once exceeding 20 cm and BER boost due to lack of the state.

Since VLC owns the directionality character, the transmission angle is also a factor to impact the transmission performance. As shown in Figure 10b, when the angle between both devices exceeds 45°, VLC is unavailable using a smartphone and TelosB sensor. The angle also affects the light intensity collected by the sensor. When the angle is less than 25°, the sensor can capture enough light intensity from the flashlight so that the VLC can work with a low BER. When the angle is between 25° and 45°, the flashlight cannot provide enough light intensity for communication, leading to boost BER.

**Figure 11:**
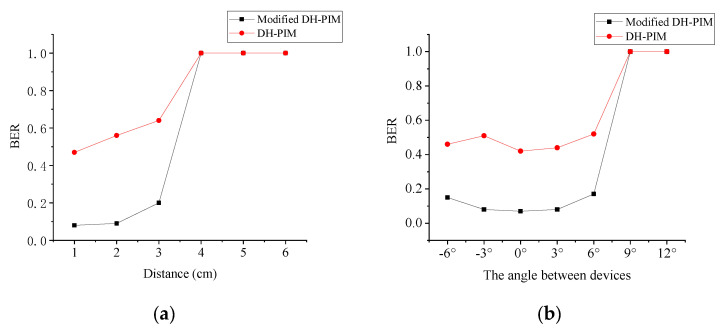
(**a**) BER under different distances in uplink. (**b**) BER under different angles in uplink.

We also use BER to measure uplink performance under different communication angles and distances, as shown in Figure 11. The distance between the camera and the sensor node determines the size of ERA in the image. From Figure 11a, modified DH-PIM obviously reduces BER when distance less than 4 cm. Once the distance is over 4 cm, uplink communication is disabled with high BER because the ERA is too small to be recognized. However, most smartphone’s cameras offer optical zoom, which can amplify the ERA and further increase communication distance.

Figure 11b shows that when the angle is smaller than 6°, the BER of modified DH-PIM can keep at a low level. When the angle is greater than 6°, the transmission cannot be performed normally. The camera cannot capture enough light to form ERA with exceeding 6° communication angle.

## 7. Conclusions

The insecure debugging interaction may lead to malicious code implantation and continuous sensitive data leakage. In this paper, we attempt to apply visible light communication in debugging interaction of sensor node and improve the security from the physical level. For this, the VLC-based hybrid communication debugging system is designed and realized with COTS devices. The system employs different visible light sensing means in uplink and downlink. For system downlink, due to the ambient light sensor’s poor performance, we employ bitmask and ODH-PIM to optimize source and channel coding for improving transmission efficiency. For the system uplink, we also optimize the symbol representation and demodulation procedure of DH-PIM to improve transmission stability of debugging feedback data. Experiments’ results demonstrate that HCDS is competent for debugging interaction of the sensor node without any hardware modification.

In future work, we will dig into the potential capacity of drone for HCDS. Object detection and route recognition techniques will be studied for HCDS with drone. By sending drone to place nearby the lost sensor nodes, it extends VLC-based debugging interaction to most outdoor applications.

## Figures and Tables

**Table 1:** Four different patterns.

Pattern	Value	Segment Size and Number of Groups
Pattern 1	00	-, -
Pattern 2	01	4 bit, 1 group
Pattern 3	10	8 bits, 2 groups
Pattern 4	11	8 bits, 2 groups

**Table 2:** Compression information.

Function Name	Brief	Size (Bit)	Compression Rate	Compression Time (s)
Timing Fun	Control timer	904	84.62%	0.47
Bubble Sort Fun	Data sorting	232	78.84%	0.21
Dijkstra Fun	Find the shortest path	1744	90.94%	0.76
LS Ctrl Fun	Control light sensor	1848	87.87%	0.84
Horspool Fun	Character match	1184	92.14%	0.56
